# Accuracy of genomic BLUP when considering a genomic relationship matrix based on the number of the largest eigenvalues: a simulation study

**DOI:** 10.1186/s12711-019-0516-0

**Published:** 2019-12-12

**Authors:** Ivan Pocrnic, Daniela A. L. Lourenco, Yutaka Masuda, Ignacy Misztal

**Affiliations:** Department of Animal and Dairy Science, University of Georgia, Athens, GA 30602 USA

## Abstract

**Background:**

The dimensionality of genomic information is limited by the number of independent chromosome segments (*M*_*e*_), which is a function of the effective population size. This dimensionality can be determined approximately by singular value decomposition of the gene content matrix, by eigenvalue decomposition of the genomic relationship matrix (GRM), or by the number of core animals in the algorithm for proven and young (APY) that maximizes the accuracy of genomic prediction. In the latter, core animals act as proxies to linear combinations of *M*_*e*_. Field studies indicate that a moderate accuracy of genomic selection is achieved with a small dataset, but that further improvement of the accuracy requires much more data. When only one quarter of the optimal number of core animals are used in the APY algorithm, the accuracy of genomic selection is only slightly below the optimal value. This suggests that genomic selection works on clusters of *M*_*e*_.

**Results:**

The simulation included datasets with different population sizes and amounts of phenotypic information. Computations were done by genomic best linear unbiased prediction (GBLUP) with selected eigenvalues and corresponding eigenvectors of the GRM set to zero. About four eigenvalues in the GRM explained 10% of the genomic variation, and less than 2% of the total eigenvalues explained 50% of the genomic variation. With limited phenotypic information, the accuracy of GBLUP was close to the peak where most of the smallest eigenvalues were set to zero. With a large amount of phenotypic information, accuracy increased as smaller eigenvalues were added.

**Conclusions:**

A small amount of phenotypic data is sufficient to estimate only the effects of the largest eigenvalues and the associated eigenvectors that contain a large fraction of the genomic information, and a very large amount of data is required to estimate the remaining eigenvalues that account for a limited amount of genomic information. Core animals in the APY algorithm act as proxies of almost the same number of eigenvalues. By using an eigenvalues-based approach, it was possible to explain why the moderate accuracy of genomic selection based on small datasets only increases slowly as more data are added.

## Background

Genomic best linear unbiased prediction (GBLUP) is a common tool for genomic analysis in animal and plant breeding [1]. Its basic form is equivalent to single nucleotide polymorphism (SNP) BLUP [2] and assumes an identical distribution of all SNP effects [1, 3, 4]. When not all the individuals are genotyped, a special version of GBLUP called single-step GBLUP (ssGBLUP) can merge pedigree and genomic relationships into a single matrix [5]. The advantage of GBLUP (and especially ssGBLUP) is simplicity, since existing models and BLUP software can be reused just by changing a relationship matrix.

GBLUP and ssGBLUP have become popular methodologies for the genetic evaluation of livestock. Although Bayesian variable selection methods [2, 6] were found to be more accurate with small datasets, their advantage seemed to be lost with large reference populations [7]. Daetwyler et al. [8] showed that selection of SNPs via BayesB outperformed GBLUP only if the number of quantitative trait loci (QTL) was small compared to the number of independent chromosome segments ($M_{e}$). Therefore, if the amount of phenotypic data is small, SNPs that are selected by tagging large QTL segments can improve accuracy by reducing the number of parameters to estimate. Karaman et al. [7] found that the advantage of BayesB over GBLUP fades with large datasets. Consequently, when the amount of information is sufficient to estimate most of the segments, selection of SNPs is no longer beneficial. Although selection of SNPs is possible with GBLUP [9, 10], its application is difficult in complex multitrait models, such as those used for commercial genetic evaluations.

There are several formulas to determine $M_{e}$. The first formula reported by Stam [11] is based on the number of chromosome junctions in a fixed size population with random mating, i.e. $4N_{e} L$, where $N_{e}$ is the effective size of the population and $L$ is the genome length in Morgan. By taking selection into account, Hayes et al. [12] reduced that number to $2N_{e} L$, and Goddard [4] reduced that number even further to $2N_{e} L/\log (4N_{e} L)$. Assuming typical values for $N_{e}$ (100) and $L$ (30) in Holstein dairy cattle, according to these three formulas, $M_{e}$ would be equal to 12,000, 6000, and 600, respectively.

Pocrnic et al. [13] related $M_{e}$ to the dimensionality of the genomic relationship matrix (GRM). For large populations that are genotyped with many SNPs, $N_{e} L$, $2N_{e} L$, and $4N_{e} L$ corresponded approximately to the number of eigenvalues that explained 90, 95, and 98% of the GRM variation, respectively. To determine which number of eigenvalues maximizes the accuracy of genomic selection, they applied ssGBLUP with a GRM inverted by the algorithm for proven and young (APY) [14], which computes a sparse generalized inverse while indirectly assuming $M_{e}$ as derived in Misztal [15]. The accuracy of prediction was maximized for a range of $N_{e}$ when the assumed dimensionality was approximately $4N_{e} L$. However, the accuracy was only marginally lower when the assumed dimensionality was $2N_{e} L$ or $N_{e} L$. Pocrnic et al. [16] found similar results when analyzing field datasets for dairy and beef cattle, pigs, and chickens and estimated the $M_{e}$ at ~ 10,000 to 15,000 in cattle and ~ 4000 in pigs and chickens. Although the theory of genomic prediction by chromosome segments is interesting, it seems to be incomplete. Assuming that all chromosome segments are independent and approximately of equal size, Daetwyler et al. [8, 17], Goddard [4], Goddard et al. [18] presented several formulas to estimate accuracy of genomic selection based on heritability, $M_{e}$, and the size of the reference population. However, in a meta-analysis using field datasets, their formulas had little predictive power [19].

If all the segments had approximately the same size, assuming half the optimal dimensionality in the APY (the largest eigenvalues that explained 98% of the GRM variation/2) would lead to half the reliability compared with using full dimensionality. However, using half of the optimal number as core animals reduced the reliability by less than 2%, and using only a third of that number reduced the reliability by less than 5% [13, 16]. Therefore, the decrease in reliability was tiny with both simulated and field datasets. In Pocrnic et al. [16], approximately 25% of the eigenvalues explained more than 90% of the genetic variation in the GRM. This suggests that genomic selection by GBLUP (and SNP BLUP) can also be seen as being based on estimates of eigenvalues of GRM. The first purpose of our study was to determine the distribution of eigenvalues in a GRM as well as the GBLUP accuracy when only the top eigenvalues of the GRM are considered. The second purpose was to determine if the optimum number of core animals in the APY algorithm is more related to the number of independent chromosome segments or to the number of top eigenvalues.

## Methods

### Data simulation

Data for this study were generated using the QMSim software [20]. Each of the simulated scenarios was replicated five times. The initial historical population consisted of 1250 generations with a gradual decrease in size from 5000 to 1000 breeding individuals and then an increase to 25,015 breeding individuals with equal sex ratio, non-overlapping generations, random mating, no selection, and no migration, in order to create a bottleneck and initial linkage disequilibrium (LD) and to establish mutation-drift balance in the population. Then, 10 discrete, recent generations with $N_{e}$ of ~ 40 were simulated by random mating of 1000 females and 10 males per generation, which resulted in 6000 genotyped individuals in generations 8 to 10. Phenotypes for individuals from generations 8 and 9 were simulated with an overall mean as the only fixed effect and with assumed heritabilities of 0.1, 0.3, 0.6, and 0.9. Scenarios with a heritability of 0.6 were replicated by simulating half (3000) and twice (12,000) the number of genotyped animals. To keep $N_{e}$ consistent across scenarios with increasing or decreasing numbers of animals, the number of breeding males per generation was fixed at 10. The simulated genome was assumed to have 10 chromosomes of equal length of 100 cM each; 3000 biallelic and randomly distributed QTL affected the trait, with allelic effects sampled from a gamma distribution as predefined in the QMSim software. The recurrent mutation rate of the markers and QTL was assumed to be 2.5 × 10^−5^ per locus per generation [21]. The first generation of the historic population had 50,000 evenly allocated biallelic SNPs with equal allele frequencies.

### Model and GRM matrices

GBLUP was used for the analysis with the following model ${\mathbf{y}} = {\mathbf{1}}\mu + {\mathbf{u}} + {\mathbf{e}}$ with ${\text{var}}\left( {\mathbf{u}} \right) = {\mathbf{G}}\sigma_{{\mathbf{u}}}^{2}$ and ${\text{var}}\left( {\mathbf{e}} \right) = {\mathbf{I}}\sigma_{{\mathbf{e}}}^{2}$, where ${\mathbf{y}}$ is a vector of phenotypes, $\mu$ is a simple mean, ${\mathbf{u}}$ is a vector of animal effects, ${\mathbf{e}}$ is a vector of residuals, ${\mathbf{G}}$ is a GRM, $\sigma_{{\mathbf{u}}}^{2}$ is the additive variance set to result in the desired heritability, and $\sigma_{{\mathbf{e}}}^{2}$ is the residual variance.

GBLUP was run with three options for the GRM. For the first option, a standard GRM was constructed as in VanRaden [1]:

$${\mathbf{G}} = \frac{{{\mathbf{ZZ}}^{ '} }}{{2\sum {p_{j} } \left( {1 - p_{j} } \right)}},$$

where ${\mathbf{Z}}$ is a matrix of allele content centered for allele frequency and $p_{j}$ is the allele frequency for marker $j$. For the second option, a reduced-rank GRM was constructed based on ${\mathbf{G}} = {\mathbf{UDU^{\prime}}}$, where ${\mathbf{U}}$ is a matrix of eigenvectors and ${\mathbf{D}}$ is a diagonal matrix of eigenvalues arranged from the highest to the lowest value. Then, a GRM restricted to $r$ eigenvalues and eigenvectors (${\mathbf{G}}_{\text{eig}}$) was constructed as ${\mathbf{G}}_{\text{eig}} = {\mathbf{UD}}_{r} {\mathbf{U^{\prime}}}$, where ${\mathbf{D}}_{r}$ includes only the $r$ largest eigenvalues in ${\mathbf{D}}$. To enable inversion in GBLUP, 0.01 ${\mathbf{I}}$ was added to both ${\mathbf{G}}$ and ${\mathbf{G}}_{\text{eig}}$ for full rank. This method is equivalent to using the largest singular values in the SNP-BLUP design matrix (${\mathbf{Z}}$). As a third option, the inverse of the GRM was derived using APY (${\mathbf{G}}_{\text{APY}}^{ - 1} )$ as in Misztal [15]:

$${\mathbf{G}}_{\text{APY}}^{ - 1} = \left[ {\begin{array}{*{20}c} {{\mathbf{G}}_{cc}^{ - 1} } & 0 \\ 0 & 0 \\ \end{array} } \right] + \left[ {\begin{array}{*{20}c} { - {\mathbf{G}}_{cc}^{ - 1} {\mathbf{G}}_{cn} } \\ {\mathbf{I}} \\ \end{array} } \right]{\mathbf{M}}_{nn}^{ - 1} \left[ { - {\mathbf{G}}_{nc} {\mathbf{G}}_{cc}^{ - 1} {\mathbf{I}}} \right],$$

where $c$ and $n$ designate core and noncore animals, respectively, in blocks of ${\mathbf{G}}$ and

$${\mathbf{M}}_{nn} = {\text{diag}}\left\{ {m_{nn,i} } \right\} = {\text{diag}}\left\{ {g_{ii} - {\mathbf{g}}_{ic} {\mathbf{G}}_{cc}^{ - 1} {\mathbf{g}}_{ci} } \right\}.$$

The inverse is sparse and requires only the dense inverse of the block of GRM for core animals.

### Computations

Standard GRM were calculated for the three populations (3000, 6000, and 12,000 genotyped animals) and replicated five times. Then, the number of eigenvalues that explained approximately 10, 30, 50, 70, 90, 95, and 98% of the variance in the GRM was computed; the fraction was defined as ${\text{tr}}\left( {{\mathbf{D}}{\text{r}}} \right)/{\text{tr}}\left( {\mathbf{D}} \right)$. Subsequent computations were performed only on the 6000-animal population. GBLUP was run using standard GRM (${\mathbf{G}})$, ${\mathbf{G}}_{\text{eig}}$, and ${\mathbf{G}}_{\text{APY}}^{ - 1}$. For ${\mathbf{G}}_{\text{APY}}^{ - 1}$, the same number of eigenvalues as for ${\mathbf{G}}_{\text{eig}}$ was used as number of core animals. Core animals were chosen randomly from all available genotypes.

### Validation

Two methods for assessing accuracy were applied. The first method calculated a realized accuracy as the correlation between the genomic estimated breeding value and the simulated breeding value for animals from the last generation without phenotypes. The second method was based on prediction error variance (PEV) that was calculated in a training set of animals. Validation was done on exactly the same animals as in the first method, but this time those animals were completely excluded from the GBLUP equations. The number of validation animals varied per scenario and was 1000, 2000, or 4000.

The accuracy for animal $i$ (${\text{acc}}_{i}$) based on PEV is calculated as follows:

$${\text{acc}}_{i} = \sqrt {1 - \frac{{{\text{PEV}}_{i} }}{{\sigma_{a}^{2} g_{ii} }}} = \sqrt {1 - \frac{{{\text{LHS}}^{ii} }}{{\sigma_{a}^{2} g_{ii} }}} ,$$

where ${\text{LHS}}^{ii}$ is the diagonal term of the inverse of the left-hand side of the mixed-model equations corresponding to animal $i$. The same accuracy can be represented as:

$${\text{acc}}_{i} \approx \sqrt {1 - \frac{\alpha }{{\alpha + d_{i}^{p} + d_{i}^{g} }}} \approx \sqrt {1 - \frac{\alpha }{{\alpha + 1 + d_{i}^{g} }}} ,$$

where $\alpha = \sigma_{e}^{2} /\sigma_{a}^{2}$ is the ratio of residual to animal genetic ($a$) variance and $d_{i}^{p}$ and $d_{i}^{g}$ are the effective number of records per individual for phenotypic and genomic information, respectively [22–24]; with one phenotype per animal, $d_{i}^{p} \approx 1$. If the amount of genomic information is calculated for animals with phenotypes only, the approximate accuracy for young animals from the same population but with no phenotypic information will be:

$$\sqrt {1 - \frac{\alpha }{{\alpha + \overline{{d_{i}^{g} }} }}} ,$$

where $\overline{{d_{i}^{g} }}$ is the average amount of genomic information based on a $d_{i}^{g}$ of a training population and is common for all the validation animals. The $d_{i}^{g}$ of a training population was based on PEV that are calculated by a direct inversion of the corresponding left-hand side of the mixed-model equation for training animals using the BLUPF90 software [25].

These two methods can be compared because they both result in a measure of accuracy based on the whole population rather than on individuals.

## Results and discussion

**Fig. 1:**
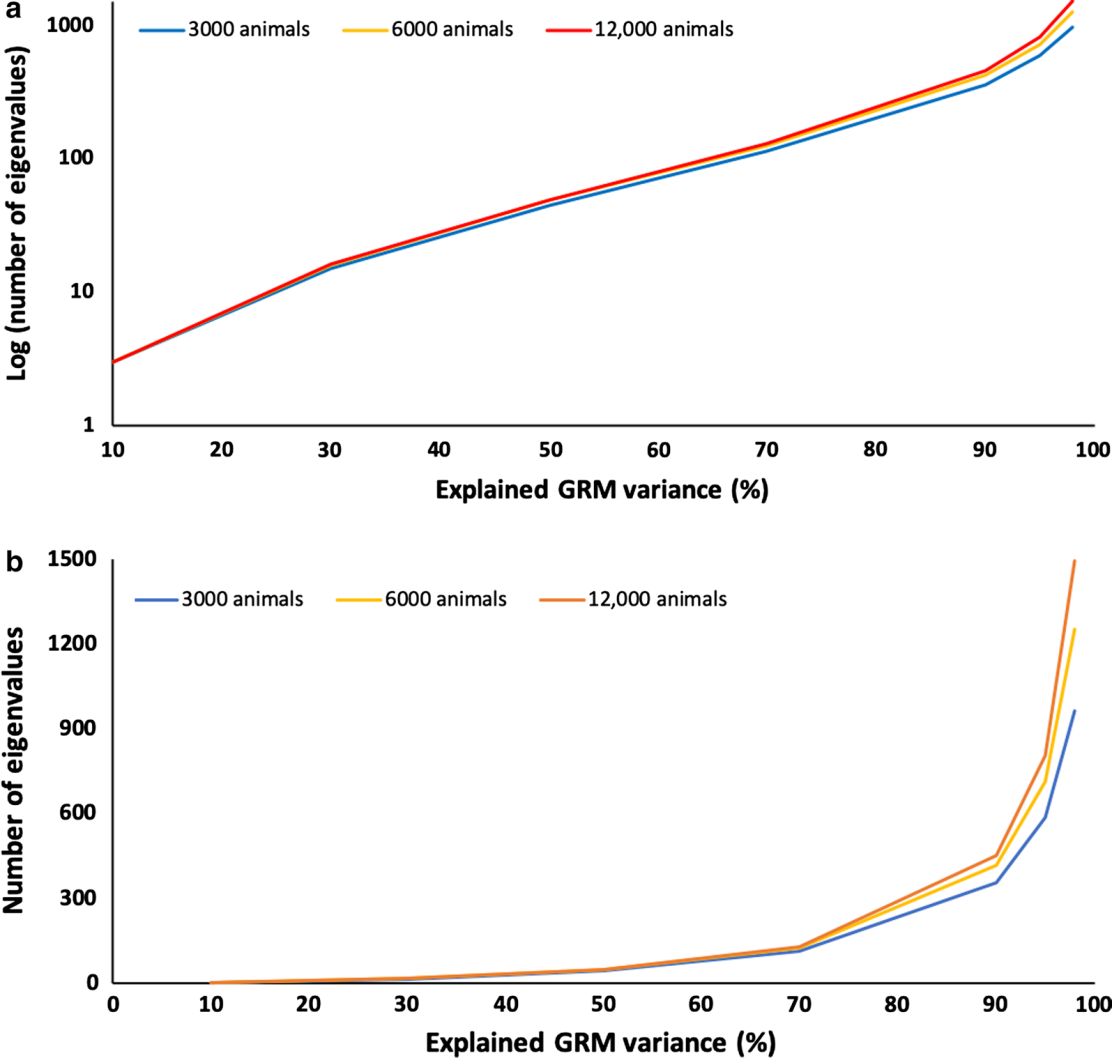
Eigenvalue profiles for explained variance of the genomic relationship matrix (GRM). Eigenvalues are expressed as either the log of the number (**a**) or the number itself (**b**) for simulated populations of 3000, 6000, and 12,000 genotyped animals

Figure 1 shows the eigenvalue profiles for 3000, 6000, and 12,000 genotyped animals. The number of eigenvalues that explained 30, 50, 70, 90, 95 and 98% of the total genomic variation ranged from 15 to 16, 45 to 49, 113 to 130, 357 to 453, 585 to 804, and 964 to 1495, respectively. Standard deviations across replicates were negligible. When varying the number of genotyped animals, the number of eigenvalues that explained a given percentage of the variance did not change much for lower percentages of explained variance, and the change was more marked for higher percentages. For lower percentages of explained variance (10 to 50%), the number of eigenvalues was relatively small (3 to 50). For higher percentages, the number of eigenvalues was more variable. For example, the number of eigenvalues that explained 90% of the GRM variance ranged from about 900 for a population of 3000 genotyped animals to 1800 for 12,000 animals. Based on Stam [11], Pocrnic et al. [13] reported that approximately $4N_{e} L$ eigenvalues explained 98% of the variance, but their study assumed a population much larger than $4N_{e} L$, and the eigenvalue profile undergoes compression at higher percentages for smaller populations. The logarithm of the number of eigenvalues explaining 30 to 90% of the GRM variance increased almost linearly.

The accuracy of GBLUP with the standard ${\mathbf{G}}$ increased with increased heritability as expected and was used as a benchmark for the ${\mathbf{G}}_{\text{eig}}$ and ${\mathbf{G}}_{\text{APY}}^{ - 1}$ methods. Average accuracy (± standard error) values were 0.69 ± 0.03, 0.79 ± 0.01, 0.90 ± 0.01, and 0.96 ± 0.00 for heritabilities of 0.1, 0.3, 0.6, and 0.9, respectively. For a heritability of 0.6 and half the number of animals (3000), average accuracy was reduced to 0.87 ± 0.01; with twice the number of animals (12,000) it increased to 0.92 ± 0.01.

**Fig. 2:**
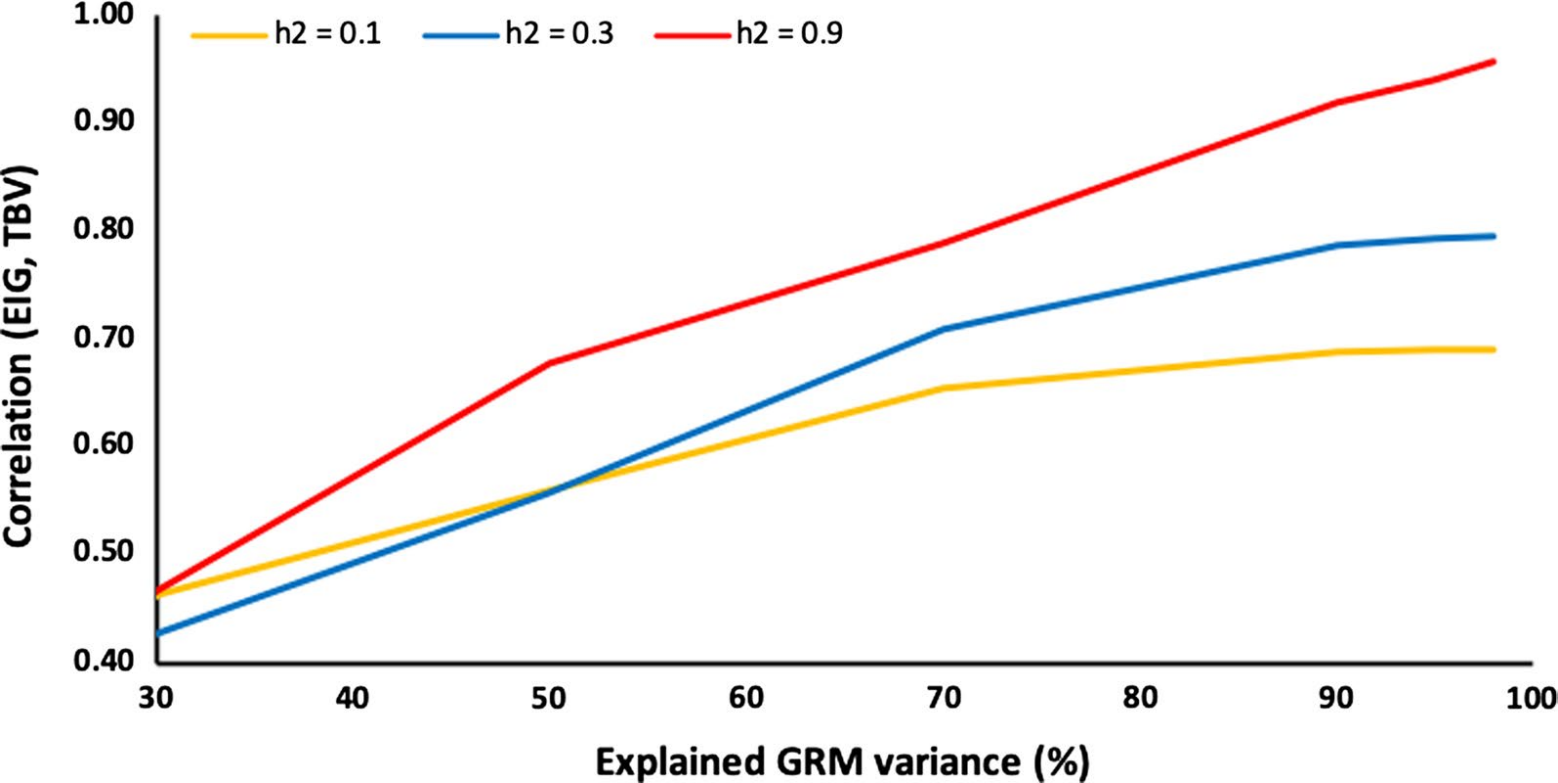
Accuracy of the genomic relationship matrix (GRM) restricted by eigenvalues based on the percentage of explained GRM variance (EIG) and heritability (*h*^2^). Accuracy is measured as the correlation between genomic estimated breeding values obtained with EIG and simulated breeding values (TBV). Heritability (*h*^2^) was 0.1, 0.3, or 0.9 for a population of 6000 genotyped animals

**Fig. 3:**
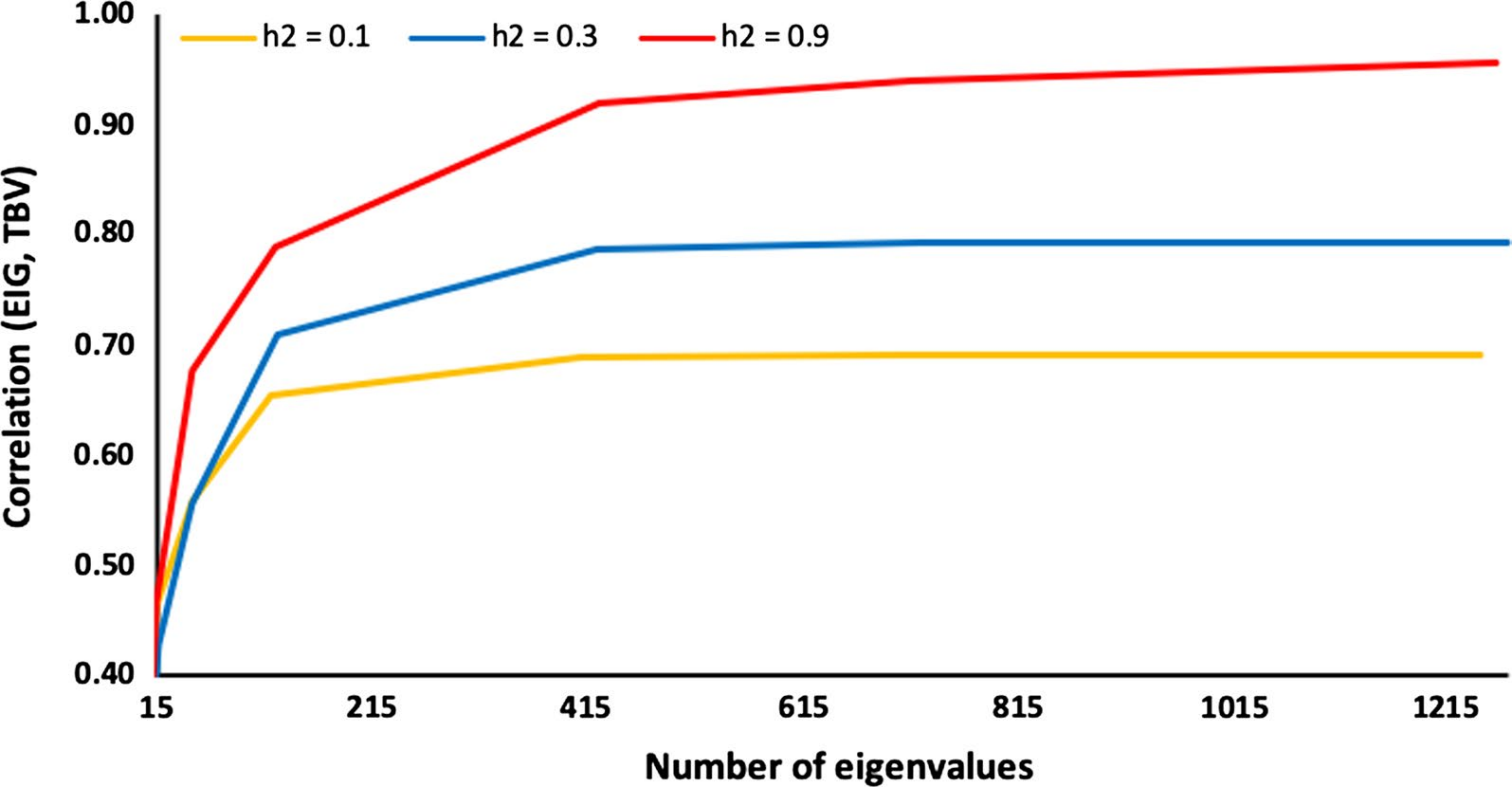
Accuracy of the genomic relationship matrix restricted by eigenvalues (EIG) based on number of eigenvalues and heritability (*h*^2^). Accuracy is measured as the correlation between genomic estimated breeding values obtained with EIG and simulated breeding values (TBV). Heritability (*h*^2^) was 0.1, 0.3, or 0.9 for a population of 6000 genotyped animals

**Fig. 4:**
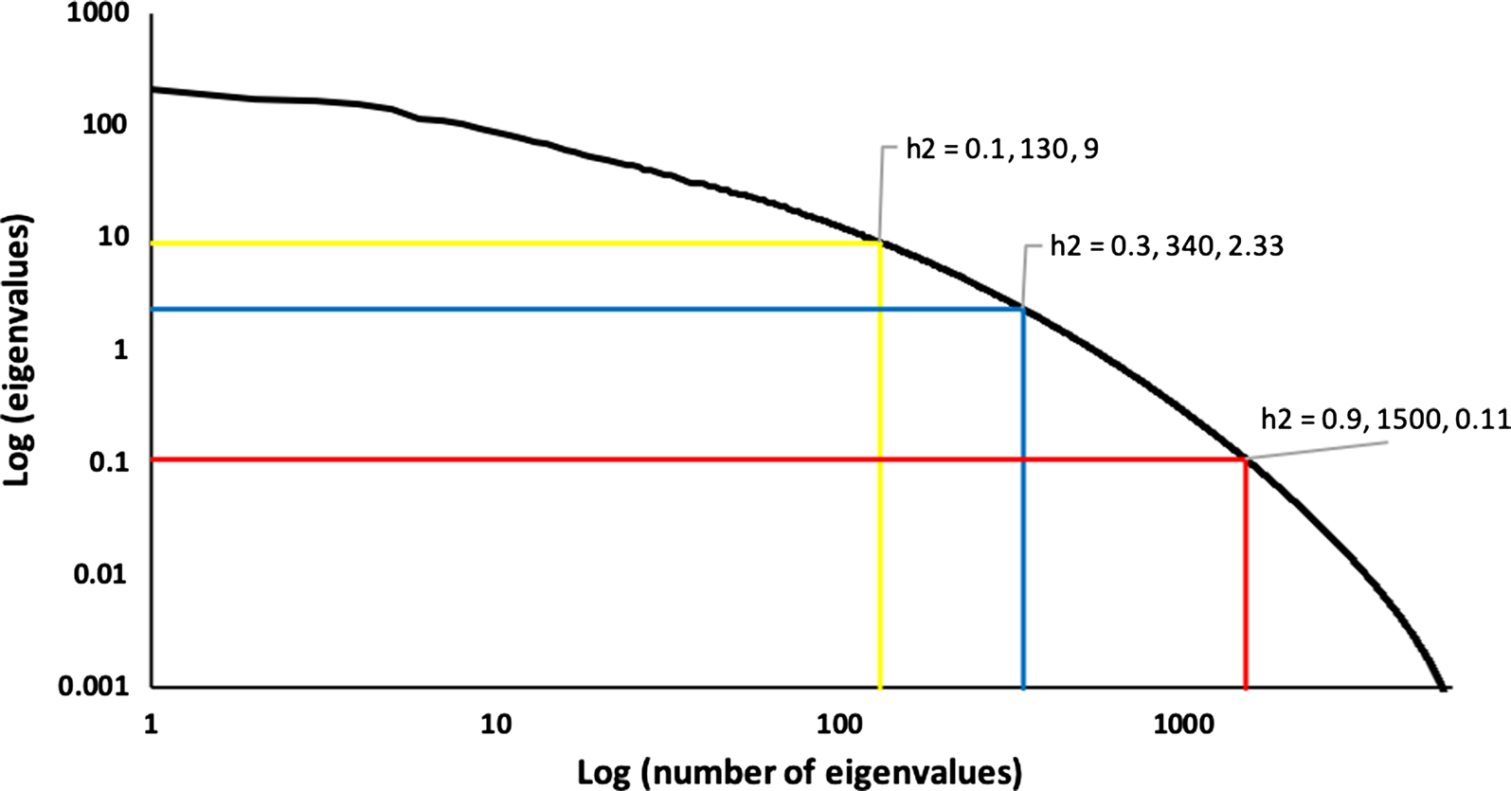
Relationship between logs of eigenvalues and numbers of eigenvalues for a population of 6000 genotyped animals. Specific curve points beyond which the eigenvalues are smaller than the ratio of residual to animal genetic variance are noted for heritabilities (*h*^2^) of 0.1, 0.3, and 0.9. The values shown after *h*^2^ are the number of eigenvalues at specific curve points and the variance ratios at given *h*^2^

The accuracy of GBLUP with ${\mathbf{G}}_{\text{eig}}$ relative to the percentage of explained GRM variance is shown in Fig. 2 and the corresponding number of eigenvalues in Fig. 3 for heritabilities of 0.1, 0.3, and 0.9 for 6000 genotyped animals. For a heritability of 0.1, accuracy stops increasing at ~ 70% of the explained variance and for a heritability of 0.3, it stops increasing at ~ 90% of the explained variance. For a heritability of 0.9, it continues to improve up to 98% of the explained variance. For all heritabilities, accuracy at 98% of the explained GRM variance was the same as for GBLUP with a standard ${\mathbf{G}}$. Figure 4 shows the eigenvalues on a logarithmic scale for 6000 genotyped animals and heritabilities of 0.1, 0.3, and 0.9 and includes points beyond which eigenvalues are smaller than the variance ratio α; details on the computation are provided in the Appendix. These eigenvalues are likely to affect accuracy, whereas smaller eigenvalues are likely to be ignored. For a heritability of 0.1, the point is approximately a log(eigenvalue) of 130, which corresponds to 70% of the explained GRM variance; the corresponding point is ~ 340 (< 90% of explained variance) for a heritability of 0.3 and ~ 1500 (98–99% of the explained variance) for a heritability of 0.9. These points correspond approximately to the points where the accuracy plateau is reached for ${\mathbf{G}}_{\text{eig}}$ (Figs. 2 and 3). The lower the heritability (or the smaller the effective information), the fewer eigenvalues are considered, and subsequently the information included in the smaller eigenvalues is ignored. With a higher heritability, the information contained in smaller eigenvalues is included.

**Fig. 5:**
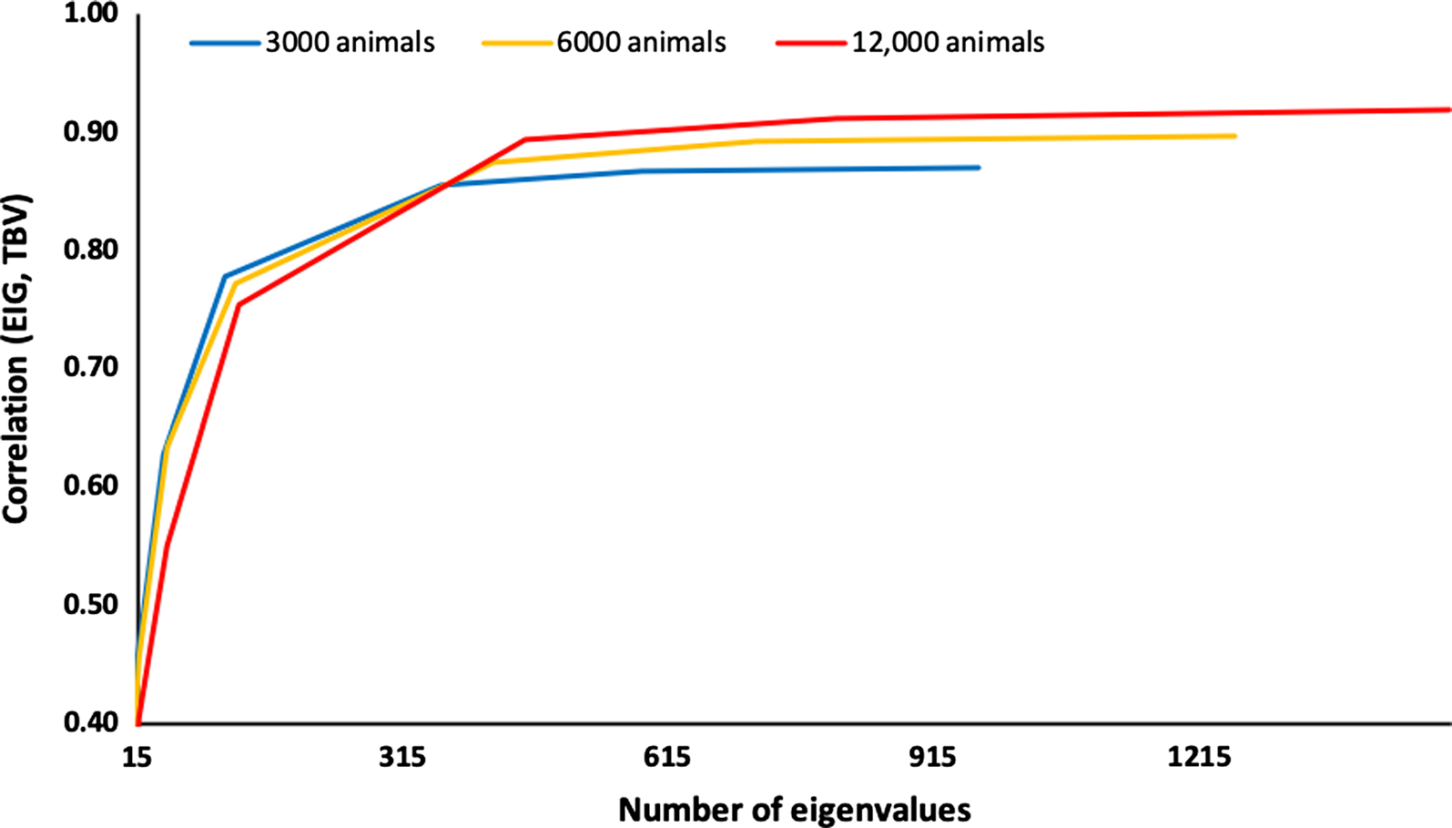
Accuracy of the genomic relationship matrix restricted by eigenvalues (EIG) based on number of eigenvalues and population size. Accuracy is measured as the correlation between genomic estimated breeding values obtained with the EIG and simulated breeding values (TBV). Population size was 3000, 6000, or 12,000 genotyped animals with a heritability of 0.6

The accuracy of GBLUP with ${\mathbf{G}}_{\text{eig}}$ relative to the number of eigenvalues is shown in Fig. 5 for population sizes of 3000, 6000, and 12,000 and a heritability of 0.6. For the largest population, accuracy is slightly lower at smaller numbers of eigenvalues and slightly higher for larger numbers of eigenvalues. In general, accuracy is expected to be higher with a larger population when a complete relationship matrix is used. However, the largest eigenvalues could correspond to the largest clusters of haplotypes, and those clusters can account for slightly more variation with smaller populations. Accuracy increases when genetically similar animals are part of the reference population; therefore, prediction accuracy for a large population with many animals for which both genotypes and phenotypes are available will improve by including additional information (e.g., herd mates) in the reference population [26]. For all population sizes, differences in accuracy were small. When the amount of phenotypic information is sufficient to estimate the effects due to most of the eigenvalues, accuracy is high and improves little with additional data.

**Fig. 6:**
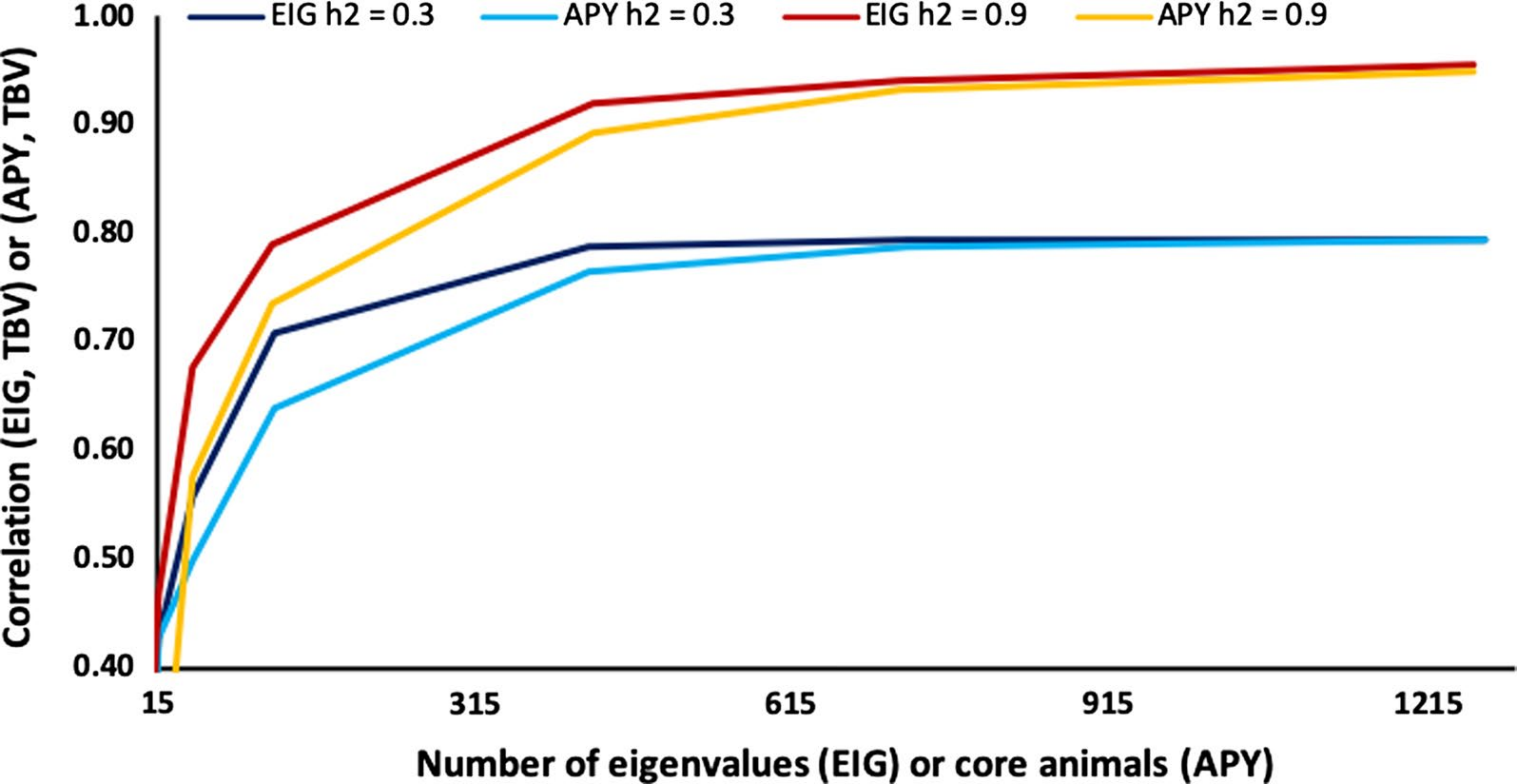
Accuracy of the genomic relationship matrix either restricted by eigenvalues (EIG) or with the inverse derived by using the algorithm for proven and young (APY) based on number of core animals [15]. Accuracy is measured as the correlation of simulated breeding values (TBV) with genomic estimated breeding values obtained with either EIG or APY. Heritability (*h*^2^) was either 0.3 or 0.9 for a population of 6000 genotyped animals

Figure 6 shows the average accuracy of GBLUP with heritabilities of 0.3 and 0.9 for ${\mathbf{G}}_{\text{eig}}$ and ${\mathbf{G}}_{\text{APY}}^{ - 1}$ using the same number of eigenvalues and core animals, respectively, for a population of 6000 genotyped animals. Accuracy is lower for ${\mathbf{G}}_{\text{APY}}^{ - 1}$ than for ${\mathbf{G}}_{\text{eig}}$ at the number of eigenvalues corresponding to 70% of the explained variance but very similar at larger numbers. Using $n$ eigenvalues is almost equivalent to assuming recursion with $n$ animals. Therefore, animal effects for any $n$ animals include almost the same information as the $n$ largest eigenvalues. Sampling variance among the five replicates was larger with ${\mathbf{G}}_{\text{APY}}^{ - 1}$ than with ${\mathbf{G}}_{\text{eig}}$, especially at smaller numbers. The choice of the core animals in the APY algorithm is critical when their number is small but not when it is large [13].

**Fig. 7:**
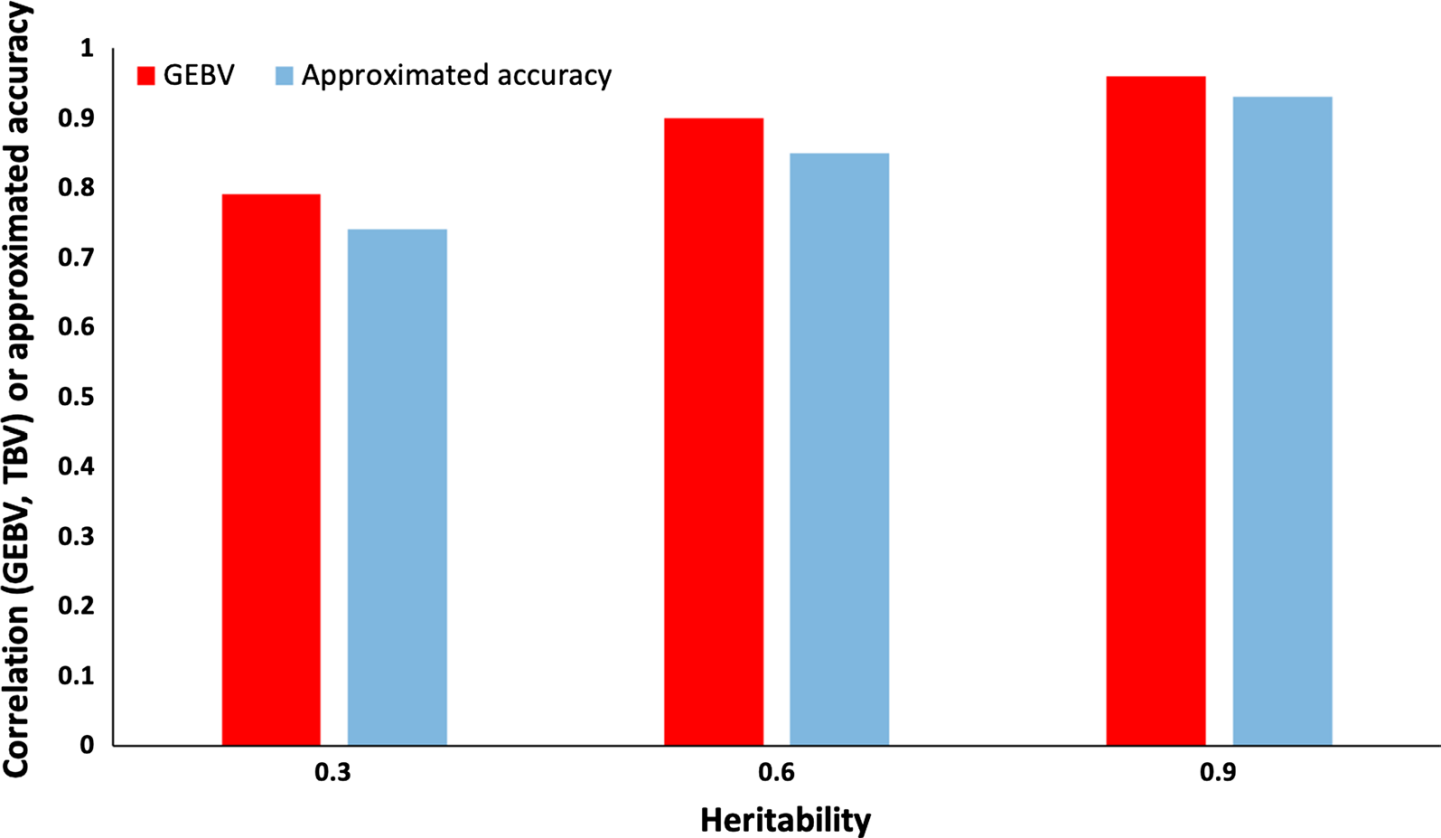
Comparison of the correlation between genomic estimated breeding values (GEBV) and simulated breeding values (TBV) with accuracy approximated from the average number of effective records. Heritability was 0.3, 0.6, or 0.9, and the simulated population included 6000 genotyped animals

Validation methods used to assess accuracy of GBLUP are compared in Fig. 7. For all heritability levels, accuracy was slightly lower for the method based on average number of effective records than for realized accuracy. The difference was largest for a heritability of 0.3 and smallest for a heritability of 0.9. The method based on average number of effective records can be a useful and simple approximation for population accuracies of validation animals.

In animal breeding programs, approximations of individual accuracy are of interest, but they cannot be derived by inversion because of the large amount of data. Although several approximations exist, those formulas are unclear when evaluations include genomic information [24, 27, 28]. One possibility is to use eigenvalue decomposition of ${\mathbf{G}}$ (possible derivations are presented in the Appendix). PEV from the direct inversion of the left-hand side of the mixed-model equation were compared with PEV from the eigenvalue decomposition of ${\mathbf{G}}$ using 2000, 4000, and 8000 genotyped animals that were treated as training animals for validation with heritabilities of 0.1, 0.3, 0.6, and 0.9. For all scenarios, correlations were equal to 1. Meuwissen et al. [29] reported a similar method for obtaining PEV using singular value decomposition for SNP BLUP. Approaches to approximate accuracy are experimental, and further research is needed to evaluate and incorporate these formulas beyond simple GBLUP, especially for ssGBLUP.

It would be useful to derive new formulas on expected genomic accuracies given the heritabilities, the number of genotyped animals and population parameters. According to this study, such an accuracy depends on the fraction of variance explained by subsequent eigenvalues. We attempted to capture that fraction given different effective population sizes and genome lengths. Preliminary studies indicated that the biggest eigenvalues were not affected by genome length, the smallest eigenvalues were affected by population size and all eigenvalues were affected by effective population size. We plan to address this issue in a future study.

## Conclusions

The distribution of eigenvalues of the GRM is very uneven, with a small fraction of the largest eigenvalues explaining a large portion of the genetic variation. The accuracy of genomic selection by GBLUP depends on how many eigenvalues can be estimated well, given the amount of information. With a small amount of information, only the effects of the largest eigenvalues are considered, but that small number of eigenvalues can explain a large portion of the genetic variation. Consequently, genomic selection is moderately accurate even with a limited amount of genomic information, and accuracy only increases slowly with larger datasets. Accuracies obtained by GBLUP using the GRM with only $n$ largest eigenvalues and corresponding eigenvectors are similar to using the APY inverse of GRM with recursion on $n$ animals. Subsequently, $n$ animals carry almost the same genomic information as the $n$ largest eigenvalues. Selection by GBLUP is based on clusters of independent chromosome segments and not on individual independent chromosome segments.

## Data Availability

The authors state that all data necessary for confirming the conclusions presented in this article are represented fully within the article. In addition, simulation parameter files are available from the corresponding author on reasonable request.

## Appendix

Derivation of PEV for individual animals using eigenvalue decomposition.

Let ${\mathbf{U}}$ be the eigenvectors and ${\mathbf{S}}$ be the eigenvalues of the GRM,

$${\mathbf{G}} = {\mathbf{USU^{\prime}}}.$$

Assuming full-rank ${\mathbf{G}}$:

$${\mathbf{G}}^{ - 1} = {\mathbf{US}}^{ - 1} {\mathbf{U^{\prime}}}.$$

The LHS of the mixed-model equation using $\alpha$ as the ratio of residual $\left( {\sigma_{{\mathbf{e}}}^{2} } \right)$ to animal genetic $\left( {\sigma_{{\mathbf{a}}}^{2} } \right)$ variance is:

$$\sigma_{{\mathbf{e}}}^{2} {\text{LHS}} = {\mathbf{I}} + \alpha {\mathbf{G}}^{ - 1} = {\mathbf{U}}\left( {{\mathbf{I}} + \alpha {\mathbf{S}}^{ - 1} } \right){\mathbf{U}}'$$

with the inverse:

$$\frac{{{\text{LHS}}^{ - 1} }}{{\sigma_{{\mathbf{e}}}^{2} }} = {\mathbf{U}}\left( {{\mathbf{I}} + \alpha {\mathbf{S}}^{ - 1} } \right)^{ - 1} {\mathbf{U^{\prime}}} = {\mathbf{UFU^{\prime}}},$$

where

$$F = {\text{diag}}\left\{ {\frac{1}{{1 + \frac{\alpha }{{s_{i} }}}}} \right\},$$

and $s_{i}$ is the eigenvalue $i$. The effect of eigenvalues on PEV is relative to the variance ratio directly and to the heritability indirectly. Such eigenvalues are especially unimportant if:

$$\frac{\alpha }{{s_{i} }} \gg 1 \to \alpha \gg s_{i} ,$$

or eigenvalues much smaller than $\alpha$ do not matter. For a high heritability, $\alpha$ is smaller and, therefore, smaller eigenvalues matter more. Individual accuracy can be computed as:

$$\frac{{{\text{LHS}}^{ii} }}{{\sigma_{e}^{2} }} = u_{i.} \left( {{\mathbf{I}} + \alpha {\mathbf{S}}^{ - 1} } \right)^{ - 1} u_{.i}^{'} ,$$

where $u_{i.}$ is the corresponding eigenvector.
